# Analysis of Lipids in Pitaya Seed Oil by Ultra-Performance Liquid Chromatography–Time-of-Flight Tandem Mass Spectrometry

**DOI:** 10.3390/foods11192988

**Published:** 2022-09-26

**Authors:** Yijun Liu, Xinghao Tu, Lijing Lin, Liqing Du, Xingqin Feng

**Affiliations:** 1 Hainan Key Laboratory of Storage & Processing of Fruits and Vegetables, Agricultural Products Processing Research Institute, Chinese Academy of Tropical Agricultural Sciences, Zhanjiang 524001, China; 2 Key Laboratory for Postharvest Physiology and Technology of Tropical Horticultural Products of Hainan Province, South Subtropical Crop Research Institute, Chinese Academy of Tropical Agricultural Sciences, Zhanjiang 524091, China; 3 Key Laboratory of Tropical Crop Products Processing of Ministry of Agriculture and Rural Affairs, Zhanjiang 524001, China; 4 College of Tropical Crops Institute, Yunnan Agricultural University, Pu’er 650201, China

**Keywords:** pitaya seed, oils, lipids, ultra-performance liquid chromatography–time-of-flight tandem mass spectrometry

## Abstract

Red pitaya (*Hylocereus undatus*) is an essential tropical fruit in China. To make more rational use of its processing, byproducts and fruit seeds, and the type, composition, and relative content of lipids in pitaya seed oil were analyzed by UPLC-TOF-MS/MS. The results showed that the main fatty acids in pitaya seed oil were linoleic acid 42.78%, oleic acid 27.29%, and palmitic acid 16.66%. The ratio of saturated fatty acids to unsaturated fatty acids to polyunsaturated fatty acids was close to 1:1.32:1.75. The mass spectrum behavior and fracture mechanism of four lipid components, TG 54:5|TG 18:1_18:2_18:2, were analyzed. In addition, lipids are an essential indicator for evaluating the quality of oils and fats, and 152 lipids were isolated and identified from pitaya seed oil for the first time, including 136 glycerides and 16 phospholipids. The main components of glyceride and phospholipids were triglycerides and phosphatidyl ethanol, providing essential data support for pitaya seed processing and functional product development.

## 1. Introduction

*Hylocereus polyrhizus*, commonly known as dragon fruit or pitaya, is native to Mexico but widely cultivated worldwide, including in southern China, Malaysia, Thailand, Vietnam, and Australia [1]. The varieties of pitaya mainly include *Hylocereus undatus, Hylocereus polyrhizus*, and *Hylocereus megalanthus* [2]. *Hylocereus undatus* is distributed primarily in tropical areas such as Guangdong and Hainan in China, and the planting area only in Guangdong has exceeded 500,000 mu. Pitaya is rich in nutrients and unique functions; it contains phytoalbumin and anthocyanins, which are rarely found in plants and are rich in vitamin C and water-soluble dietary fibers [3,4,5].

Pitaya seeds are black sesame-like kernels embedded in the flesh of pitaya; the number of seeds in a pitaya is approximately 5000–15,000, and the fruit seeds account for 1.3–1.5% of the fresh fruit weight [6]. Researchers worldwide have focused on pitaya seeds’ fatty acid composition, amino acid composition, and functionality. Pitaya seeds contain up to 32.02% fat, 22.06% protein, 21.03% starch, 1.75% total sugar, 10.35% crude fiber, and 3.18% ash [7,8,9]. The red pitaya seeds contained 6.48% moisture, 24.84% crude protein, 31.79% crude fat, 13.38% crude fiber, 2.58 g/100 g ash, and 20.39 g/100 g carbohydrate, in addition to α-tocopherol and γ-tocopherol, with 5.78 mg/100 g and 10.50 mg/100 g, respectively [10]. Pitaya seed oil’s unsaturated fatty acid content was as high as 78.10%, including 44.29% linoleic acid and 31.75% oleic acid [11]. The iodine uptake value, saponification value, and free fatty acid value of red dragon fruit seed oil were 105.6 g I_2_/100 g, 235.7 mg KOH/g, and 1.9 mg KOH/g, respectively [12]. Lim et al. [13,14] identified seven phenolic acids from pitaya seed oil with a total tocopherol content of 36.70–43.50 mg/100 g, and the phytosterol compounds identified in the *H. undatus* seed oil (WFSO) and *H. polyrhizus* seed oil (RFSO) were cholesterol, campesterol, stigmasterol, and β-sitosterol. Seven phenolic acid compounds were identified in the WFSO and RFSO: gallic, vanillic, syringic, protocatechuic, *p*-hydroxybenzoic, *p*-coumaric, and caffeic acids. WFSO and RFSO can be differentiated by their T off and T on values in the DSC thermal curves. Hao et al. [15] investigated the protein profile of pitaya fruit seeds and focused on the most reactive proteins against immunoglobulin E (IgE) in sera from allergic patients by immunoblotting. The potential allergens included cupin_1 and heat shock protein 70 (HSP70) in white-fleshed pitaya seeds, and cupin_1, heat shock protein 70, and heat shock protein sti1-like in red-fleshed pitaya seeds.

A pressing method (including a hot pressing and cold pressing method), water enzyme method, leaching method (including ultrasonic aids, etc.), the supercritical extraction method, and other methods are more commonly used in the extraction of vegetable oils and fats, including the cold pressing method using low-temperature oil presses on the raw material of oil without steaming or rolling embryos for pure physical mechanical pressing. The temperature is below 65 °C, and the resulting oil has good antioxidant and other properties [16,17,18,19]. Under the conditions of supercritical CO_2_ extraction with a crushing degree of 40 mesh, a filling capacity of 400 g (1 L extraction kettle), an extraction pressure of 25 MPa, an extraction temperature of 40 °C, and an extraction time of 3.5 h, Wang et al. [20,21] obtained a 30.21% oil yield from dragon fruit seed oil and found that the relative contents of squalene and retinal in pitaya seed oil were as high as 6.76% and 2.95%, respectively. Deng et al. [22] achieved more than 31% oil yield under supercritical extraction conditions with a CO_2_ flow rate of 20 L/h, an extraction pressure of 30 MPa, an extraction temperature of 55 °C, and an extraction time of 3 h. Wu et al. [23] used microwave-assisted hexane extraction of pitaya seed oil with a power of 495 W, a solvent ratio of 1:12, and a time of 180 s to obtain a yield of 31% pitaya seed oil. The extraction rate of pitaya seed oil was 31.47% under the conditions of a liquid-to-material ratio of 7:1 (mL:g), extraction temperature of 78 °C, extraction time of 3 h, and extraction solvent of petroleum ether by Deng et al., and the indices of acid value and peroxide value of dragon fruit seed oil reached the national standard of edible fats and oils [24].

Lipids play an essential physiological function in the growth of plants and animals and are closely related to human metabolism, among others. Xu et al. [25] determined that pitaya seed oil had a significant and sustained inhibitory effect on the proliferation of HepG-2 and HT-29 human cancer cells using the thiazole blue (MTT) assay. In addition, health problems caused by excessive consumption of edible oils and excessive fat intake have become essential factors affecting the health of our residents. The different compositions of fats and oils not only influenced their digestive and absorption effects in the human gastrointestinal tract but also affected intestinal homeostasis due to the imbalance of their intake, leading to intestinal dysfunction, which in turn induced chronic diseases such as obesity, diabetes, and hypertension, ultimately affecting human health [26]. Previous lipid analysis studies in pitaya seed oil only analyzed the composition, and more detailed information and chemical structures of lipids are needed. However, little has been reported on the whole-lipid characterization of pitaya seed oil. It is well known that analyzing the content, composition, and structure of whole pitaya seed oil lipid components is necessary. This study aimed to characterize the lipid composition of pitaya seed oil based on ultra-performance liquid chromatography–time-of-flight tandem mass spectrometry (UPLC-TOF-MS/MS) and provide data support for the application of pitaya seed oil in the food, pharmaceutical, and daily cosmetics industries.

## 2. Materials and Methods

### 2.1. Sample Preparation

Red pitaya seeds, provided by Guangdong Meichen Biotechnology Co.(Zhanjiang, China), were taken and placed in a single-screw oil press (OP101, Shenzhen Yimecom E-commerce Co., Shenzhen, China). The gross oil of pitaya seeds, made by cold pressing mode with temperature below 60 °C, was centrifuged at 5000 r/min for 10 min, and the upper oil sample was sealed and stored at 4 °C.

### 2.2. Determination of Fatty Acid Composition by Gas Chromatography

The fatty acid composition of pitaya seed oil was determined by the external standard method (Carbon XVII fatty acid methyl ester standard, Avanti Polar Lipids, U.S. Alabama) of potassium hydroxide methylation. Weigh 6–10 mg of pitaya seed oil accurately and 50 μL of 5 mg/mL of the internal standard carbon XVII fatty acid methyl ester, add 2 mL of 0.4 mol/L KOH methanol solution, vortex, and shake for 15 min, add 1 mL of n-hexane and 2 mL of 0.9% NaCl aqueous solution, shake for 2–3 s, centrifuge at 4500 rpm at 4 °C for 10 min, take the supernatant and transfer it to a 2 mL injection vial.

Gas chromatography-mass spectrometry (GC–MS) was equipped with a hydrogen flame ionization detector and DB-FastFAME column (30 m × 0.25 mm × 0.25 µm, 7890A gas chromatograph tandem hydrogen flame ionization detector, Agilent, Palo Alto, CA, U.S.). The measurement conditions were as follows: nitrogen as the carrier gas, injection volume of 1.0 μL, the inlet temperature of 260 °C, splitting ratio of 20:1; programmed temperature rise, and column initial temperature of 150 °C. The column was ramped up to 210 °C at 10 °C/min and held for 8 min, then ramped up to 230 °C at 20 °C/min and maintained for 6 min, and the detector temperature was 280 °C.

### 2.3. Determination of Lipid Composition Using UPLC-TOF-MS/MS [16]

An aliquot of 150–200 mg of pitaya seed oil was placed in a 10 mL test tube, and 10 μL of 10 μg/mL triglyceride deuterium (internal standard), 2 mL of hexane, 2 mL of methanol, and 0.2 mL of ultrapure water were added sequentially, shaken and mixed, vortexed, and centrifuged. The supernatant was removed, and the supernatant was placed in a test tube and blown dry with a nitrogen-blowing instrument (DC-24, Shanghai Ampli Experimental Technology Co., Shanghai, China). Then, 100 μL of methanol was added for re-dissolution, and the re-dissolved solution was passed through a 0.22 μm organic filter membrane and placed in a sample bottle for use.

### 2.4. Analytical Conditions for UPLC–MS

The analytical instrument was a Shimadzu UPLC LC-30A system (LC-30A liquid chromatograph, Shimadzu Corporation, Kyoto, Japan) equipped with a Phenomenex Kinete C18 column (100 × 2.1 mm, 2.6 µm). A sample of 1 µL was pumped onto the column at a rate of 0.4 mL/min. The column temperature was 60 °C, and the sample chamber temperature was 4 °C. Gradient elution was performed using phase A (H_2_O–methanol–acetonitrile = 1:1 psi1, containing 5 mM ammonium acetate) and phase B (isopropanol–acetonitrile = 5:1, containing 5 mM ammonium acetate) with elution conditions of 20% B for 0.5 min, 40% B for 1.5 min, 60% B for 3 min, 98% B for 13 min, 20% B for 13 min, and 20% B for 17 min. In addition, the mass spectrometry system (Q-TOF-6600 Mass Spectrometer, AB Sciex, Concord, Ontario, Canada) was an AB Sciex TripleTOF^®^ 6600 with an ESI ion source in positive and negative modes, and the mass number range for mass spectrometry was *m*/*z* 100–1200:50.00. The ion spray voltage was set to 5500 V (+) and −4500 V (−). Pressures of ion source gas 1, ion source gas 2, and curtain gas were set at 50, 50, and 35 psi, respectively, and the interface heater temperature was 600 °C. The detection limit of the instrument was 6 (peak height) and the quantification limit was 10.

### 2.5. Data Processing

Qualitative analysis of shotgun-MS data was performed using the LipidView software (v2.0, ABSciex, Concord, ON, Canada). Software parameter settings: mass tolerance = 0.5, min % intensity = 1, minimum S/N = 10, flow injection average spectrum from top = 30% TIC, total double bonds ≤ 12.

## 3. Results and Discussion

### 3.1. Analysis of Fatty Acid Composition in Pitaya Seed Oil

As shown in Table 1, the highest yield of 31.79% pitaya seed oil was obtained by the Soxhlet extraction method for the same species. Pitaya seed oil was mainly composed of unsaturated and saturated fatty acids, of which unsaturated fatty acids were more than 75%. Its fatty acid composition was mainly linoleic acid (40%), oleic acid (23%), and palmitic acid (15%). Linoleic acid plays a vital role in lipid metabolism in enzyme activity, the central nervous system, and other critical activities [26]. Its linoleic acid content is higher than that of canola oil (20.4%) [27], peanut oil [28], cashew nut oil (19.69%) [29], and avocado oil (6–10%) [30], which is another way to obtain linoleic acid. Because the human body cannot synthesize it, it can only be obtained from exogenous foods. Accordingly, pitaya seed oil could be added to dietary supplements or functional foods.

Extraction methods and varieties affected the seed oil’s extraction rate and fatty acid composition. The unsaturated fatty acid content of pitaya seed oil measured in this study (75.43%) was higher than that of Wang et al. (74.64%) [20] and lower than the results of Lim et al. (77.22~82.01%) [13] and Li et al. (78.10%) [11], which might stem from the different extraction methods and some other differences. In addition, linoleic acid in white pitaya seed oil was higher than that in red pitaya seed oil, and the content of the red pitaya seed oil obtained by chloroform–methanol extraction was greater than that obtained by the Soxhlet extraction. Compared with the cold pressing method, the Soxhlet and chloroform–methanol extraction methods yielded more fatty acid species in pitaya seed oil. The Soxhlet extraction method produced the most species. The extraction method affected pitaya seed oil’s extraction rate and fatty acid composition; pitaya seed oil’s composition and content varied greatly by species.

### 3.2. Identification of Lipids

After the selected lipid molecule precursor ions entered the mass spectrometry Q2, at certain collision energy (CE), the lipid molecule precursor ions underwent collision-induced dissociation (CID), and the lipid molecule produced specific fragment ions or neutral loss of specific functional groups, thus producing diagnostic ion science [31]. This study used UPLC–MS/MS to analyze and identify glycerolipids and phospholipids in pitaya seed oil. In the following research, TG 54:5|TG 18:1_18:2_18:2 in triglyceride (TG), DG 36:4|DG 18:2_18:2 in diacylglycerol (DG), phosphatidyl ethanol (PEtOH) PEtOH 34:1|PetOH 16:0_18:1, and PG 15:0_18:1 in phosphatidyl glycerol (PG) were used as examples to analyze their mass spectrometric behavior and fracture mechanisms in detail.

**Figure 1:**
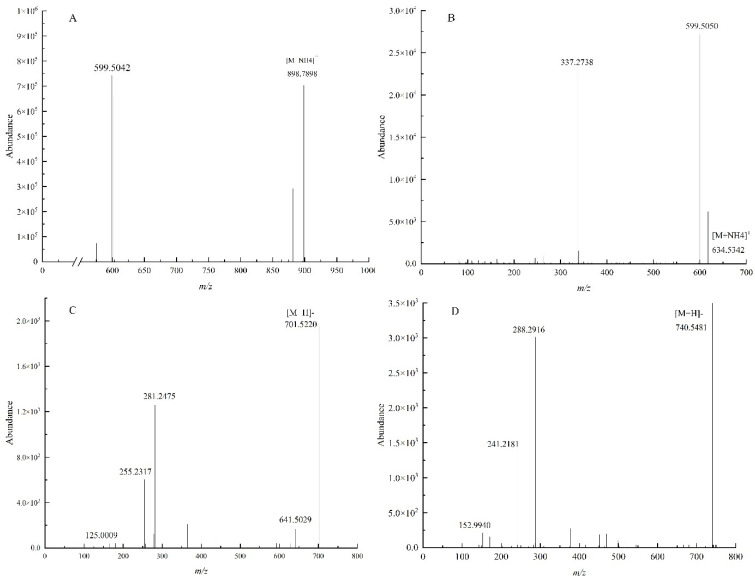
MS/MS spectra of TG 54:5|TG 18:1_18:2_18:2 (**A**) and DG 36:4|DG 18:2_18:2 (**B**) in positive ion mode and PEtOH 34:1|PEtOH 16:0_18:1 (**C**) and PG 15:0_18:1 (**D**) in negative ion mode.

The molecular species of compounds were identified by retention time, isotopic distribution, MS mass-to-charge ratio, and MS/MS secondary mass spectrometry in positive and negative ion modes. Figure 1A shows the MS/MS spectra of TG 54:5|TG 18:1_18:2_18:2 in positive ion mode. From Figure 1A, *m*/*z* 898.7898 was the [M + NH_4_]^+^ parent ion of this compound, which was neutral to the loss of FA 18:1 and FA 18:2 to produce two diester fragment ions, which were 36:4DDAG + (*m*/*z* 599.5042) and 36:3DDAG + (*m*/*z* 601.5217), respectively. Figure 1B shows the MS/MS spectra of DG 36:4|DG 18:2_18:2 in the positive ion mode. Figure 1B shows that *m*/*z* 634.5342 could be tentatively determined as [M + NH_4_] + for DG 36:4. *m*/*z* 599.5050 was [M + NH_4_-NH_3_-H_2_O]^+^, which was the fragment ion formed by the [M + NH_4_] + parent ion after the neutral loss of NH_3_ and H_2_O. *m*/*z* 337.2728 was the fatty acid acyl chain characteristic diagnostic fragment ion ([M + NH_4_-NH_3_-FA 18:2]^+^), which was the monoglyceride fragment ion 18:2 DMAG+ formed after the loss of one fatty acid FA 18:2 by the parent ion *m*/*z* 634.5342. Figure 1C shows the MS/MS spectrum of PEtOH 34:1|PEtOH 16:0_18:1 in negative ion mode. As shown in Figure 1C, *m*/*z* 701.5220 was the [M-H]^−^ parent ion of PEtOH 34:1, *m*/*z* 125.0009 was phosphoethanol, and *m*/*z* 255.2317 and *m*/*z* 281.2475 were [FA 16:0-H]^−^ and [FA 18:1-H]^−^, respectively. Figure 1D shows the MS/MS spectrum of PG 15:0_18:1 in negative ion mode. As shown in Figure 1D, *m*/*z* 740.5481 was the [M-H]^−^ parent ion, *m*/*z* 241.2181 and *m*/*z* 288.2916 were [FA 15:0-H]^−^ and [FA18:1-H]^−^, respectively, and *m*/*z* 152.9940 was [phosphoglycerol-H_2_O-H]^−^, which was the loss of a water molecule from the phosphoglycerol hydrogen peak.

### 3.3. Analysis of Lipid Composition in Pitaya Seed Oil

**Figure 2:**
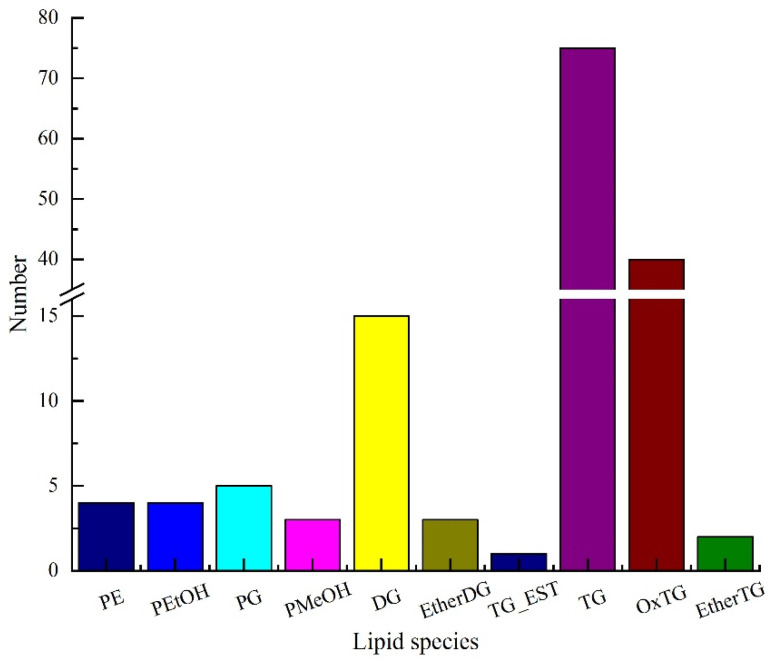
Lipids of pitaya seed oil.

The lipid composition of pitaya seed oil was comprehensively profiled using UPLC-TOF-MS/MS. Information on the precise relative molecular mass, isotopic distribution, and secondary mass spectrometry cleavage fragmentation of the lipids was obtained using the composite scan mode. As shown in Figure 2, 152 lipids were identified in pitaya seed oil, of which 136 were glycerides and 16 were phospholipids. The 136 glycerides mainly included 15 diacylglycerols (DG), three ether-linked diacylglycerols (EtherDG), one triacylglycerol estolide (TG_EST), 75 triglycerides (TG), 40 oxidized triglycerides (OxTG), and two ether-linked triacylglycerols (EtherTG). The 16 phospholipids included four phosphatidylethanolamines (PE), four phosphatidyl ethanols (PEtOH), five phosphatidyl glycerols (PG), and three phosphatidyl ethanols (PMeOH).

As shown in Table 2, the total number of carbon atoms in the fatty acid side chains of lipids in pitaya seed oil was 32–71, and the double bond number was 0–7. Most lipids contained at least one fatty acid side chain with a carbon number of 18 and a double bond number of 0–3. The number of carbon atoms of DG in glycerides was 32–40, with a double bond number of 0–5 and a C18 side chain in each lipid. The number of carbon atoms of EtherDG was 34–36, and the number of double bonds was 2–4. The number of carbon atoms of TG_EST was 71, with six double bonds, and consisted mainly of three carbon chains, C15, C18, and C22, of which the C18 and C22 side chains contained 2 and 3 carbon-carbon double bonds, respectively. The number of carbon atoms of TG was 34–62, and the double bond number was 0–7. The number of carbon atoms of OxTG was 41–58, and the number of double bonds was 0–6. The number of carbon atoms of EtherTG was 55–59, and the number of double bonds was 3–5. The number of carbon atoms of PE in phospholipids was 34–42, and the double bond number was 1–4. The number of carbon atoms of PEtOH was 34–36, and the number of double bonds was 1–4. The number of carbon atoms of PG was 29–42, and the number of double bonds was 0–4. The number of carbon atoms of PMeOH was 16–34, and the double bond number was zero.

### 3.4. Analysis of the Lipid Content of Pitaya Seed Oil

**Figure 3:**
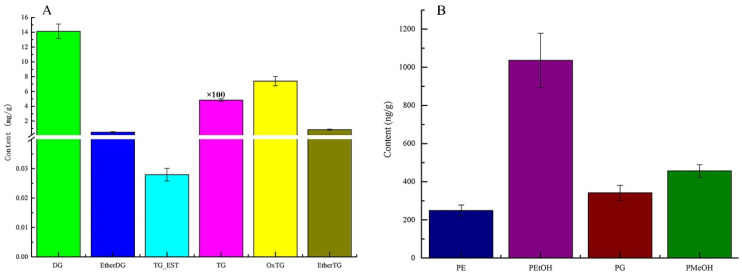
The composition of phospholipids (**A**) and glycerides (**B**) in pitaya seed oil. TG content is multiplied by 100 times.

Under the same conditions, the mass spectra of the same class of lipids should be similar and comparable. In this experiment, the peak areas of the extracted ion chromatographic peaks in the primary mass spectra of pitaya seed oil were used for the quantitative calculation of the same class of lipids, as shown in Figure 3. As seen in Figure 3, the lipid composition of pitaya seed oil mainly consisted of glycerides and phospholipids with contents of 505.69 ± 18.79 mg/g and 2.08 ± 0.24 mg/g, respectively. Glycerides were mostly TG, DG, and OxTG, with contents of (482.80 ± 17.0) mg/g, (7.41 ± 0.64) mg/g, and (14.12 ± 0.98) mg/g, accounting for 95.47, 2.79, and 1.46% of the glycerides, respectively. Thus, these three compounds accounted for 99.73% of the total glycerides. Dietary triglycerides are the main component of oils, and their main functions are to supply and store energy, fix and protect internal organs, participate in the energy supply in several aspects of maternal and intrauterine fetal growth and development during pregnancy, and play a key role in lipid metabolism [32,33]. Accordingly, pitaya seed oil could be used in oil and fat dietary supplements.

Phospholipids were mainly PEtOH (1.04 ± 0.14 mg/g), accounting for 49.74% of the total phospholipid content. In oilseeds, phospholipids are primarily present in the colloidal phase in a complex state with molecules such as proteins and sugars [34]. In general, phospholipids in plant oilseeds consist mainly of PC, PE, and PI, and the content of phospholipids varies from one oilseed to another or from one variety and growing region of the same oilseed [35]. While PEtOH and PMeOH are the main components of dragon fruit seed oil, there are significant differences in lipid composition among soybean, sesame, peanut, and rapeseed [36]. The phospholipids in food are transformed into choline through a series of chemical reactions under the body’s digestive action, which can promote the speed of information transmission between nerve cells in the brain and enhance memory function. In addition, phospholipids not only improved arterial vascular composition, but also maintained esterase activity, improved lipid metabolism in the body, emulsified neutral esters and cholesterol deposited in the vascular wall, promoted the absorption of fats and fat-soluble vitamins, and enhanced intelligence and cellular activity [37,38]. Therefore, pitaya seed oil has broad application prospects in functional product development.

## 4. Conclusions

In this study, the lipid profile of pitaya seed oil was first profiled by UPLC-TOF-MS/MS, and 11 fatty acid components were identified from pitaya seed oil, mainly linoleic acid, oleic acid, and palmitic acid, with an unsaturated fatty acid content of more than 75%, which are highly unsaturated fatty acid oils. In addition, 152 lipid components were identified in pitaya seed oil, mainly composed of 136 triglycerides and 16 phospholipids, with triglyceride content (505.69 ± 18.79) mg/g, which provided basic data support for the later separation of pitaya seed components and in-depth functional research.

## Figures and Tables

**Table 1:** Fatty acid composition in pitaya seed oil (%).

Fatty Acid	Red Pitaya Seed			White Pitaya Seed
	Cold Pressing Method	Chloroform–Methanol Extraction [10]	Soxhlet Extraction [10]	Soxhlet Extraction [6]
C12:0	ND	ND	0.13 ± 0.01	ND
C13:0	ND	ND	0.04 ± 0.02	ND
C14:0	0.12 ± 0.00	0.22 ± 0.01	0.39 ± 0.13	0.30 ± 0.01
C16:0	16.66 ± 0.02	15.85 ± 0.12	16.64 ± 0.68	17.10 ± 0.78
C16:1	0.66 ± 0.00	0.82 ± 0.04	1.01 ± 0.22	0.61 ± 0.01
C17:0	ND	0.08 ± 0.00	0.10 ± 0.02	ND
C18:0	4.78 ± 0.02	5.24 ± 0.36	6.05 ± 0.09	4.37 ± 0.24
C18:1	27.29 ± 0.07	26.66 ± 0.27	26.63 ± 0.09	23.80 ± 0.14
C18:2 n6t	4.15 ± 0.05	3.91 ± 0.30	3.97 ± 0.12	2.81 ± 0.10
C18:2 n6c	42.78 ± 0.05	43.12 ± 1.16	39.76 ± 1.23	50.10 ± 0.35
C18:3 n3	0.29 ± 0.00	0.43 ± 0.04	0.59 ± 0.19	0.98 ± 0.10
C20:0	1.48 ± 0.01	1.39 ± 0.07	1.44 ± 0.10	ND
C20:1	0.25 ± 0.09	0.29 ± 0.01	0.37 ± 0.08	ND
C22:0	1.54 ± 0.03	1.25 ± 0.05	1.35 ± 0.09	ND
C23:0	ND	0.48 ± 0.04	1.12 ± 0.15	ND
C24:0	ND	0.26 ± 0.01	0.41 ± 0.02	ND
Saturated fatty acids	24.57 ± 0.08	24.77 ± 0.57	27.67 ± 0.75	21.70 ± 1.03
Monounsaturated fatty acids	32.36 ± 0.21	31.39 ± 0.57	31.61 ± 0.20	27.20 ± 0.25
Polyunsaturated fatty acids	43.07 ± 0.05	43.84 ± 1.12	40.72 ± 0.95	51.10 ± 0.45
Total unsaturated fatty acids	75.43 ± 0.36	75.23 ± 1.69	72.33 ± 1.15	78.30 ± 0.70
Oil extraction rate (g/100 g seeds)	19.48 ± 1.04	19.35 ± 1.36	31.79 ± 1.19	32.00 ± 1.40

Note: ND means “not detected”, C12:0—lauric acid, C13:0—tridecanoic acid, C14:0—myristic acid, C16:0—palmitic acid, C16:1—palmitoleic acid, C17:0—heptadecanoic acid, C18:0—stearic acid, C18:1—oleic acid, C18:2 n6t—linolelaidic acid, C18:2 n6c—linoleic acid, C18:3 n3—α-linolenic acid, C20:0—arachidic acid, C20:1—cis-11-eicosenoic acid, C22:0—behenic acid, C24-0—lignoceric acid.

**Table 2:** Composition of the 152 lipids in pitaya seed oil.

No	Lipid Species	Fatty Acid Composition (Carbon Number: Double Bond Number)	No	Lipid Species	Fatty Acid Composition (Carbon Number: Double Bond Number)
1	PE	PE 34:2|PE 17:1_17:1	25	DG	DG 36:5|DG 18:2_18:3
2	PE	PE 36:2|PE 18:0_18:2	26	DG	DG 38:1|DG 20:0_18:1
3	PE	PE 39:4|PE 18:1_21:3	27	DG	DG 38:2|DG 20:0_18:2
4	PE	PE 42:1; O|PE 16:1_26:0; O	28	DG	DG 38:3|DG 20:1_18:2
5	PEtOH	PEtOH 34:1|PEtOH 16:0_18:1	29	DG	DG 40:1|DG 22:0_18:1
6	PEtOH	PEtOH 36:1|PEtOH 18:0_18:1	30	DG	DG 40:2|DG 22:0_18:2
7	PEtOH	PEtOH 36:2|PEtOH 18:1_18:1	31	DG	DG 40:3|DG 22:1_18:2
8	PEtOH	PEtOH 36:4|PEtOH 18:2_18:2	32	EtherDG	DG O-34:2|DG O-17:0_17:2
9	PG	PG 29:1|PG 10:0_19:1	33	EtherDG	DG O-36:3|DG O-19:1_17:2
10	PG	PG 33:1; O|PG 16:0_17:1; O	34	EtherDG	DG O-36:4|DG O-19:2_17:2
11	PG	PG 34:2|PG 16:0_18:2	35	TG_EST	TG 71:6; O2|TG 15:0_18:2 22:3; O (FA 16:0)
12	PG	PG 35:0|PG 16:0_19:0	36	TG	TG 34:0|TG 8:0_10:0_16:0
13	PG	PG 42:4|PG 21:2_21:2	37	TG	TG 36:0|TG 10:0_12:0_14:0
14	PMeOH	PMeOH 16:0|PMeOH 8:0_8:0	38	TG	TG 36:1|TG 8:0_10:0_18:1
15	PMeOH	PMeOH 32:0|PMeOH 16:0_16:0	39	TG	TG 38:0|TG 8:0_14:0_16:0
16	PMeOH	PMeOH 34:0|PMeOH 15:0_19:0	40	TG	TG 38:1|TG 10:0_10:0_18:1
17	DG	DG 32:0|DG 16:0_16:0	41	TG	TG 40:0|TG 10:0_14:0_16:0
18	DG	DG 34:0|DG 16:0_18:0	42	TG	TG 40:1|TG 8:0_14:0_18:1
19	DG	DG 34:1|DG 16:0_18:1	43	TG	TG 42:0|TG 10:0_16:0_16:0
20	DG	DG 34:3|DG 16:1_18:2	44	TG	TG 42:1|TG 8:0_16:0_18:1
21	DG	DG 36:1|DG 18:0_18:1	45	TG	TG 42:2|TG 8:0_16:0_18:2
22	DG	DG 36:2|DG 18:1_18:1	46	TG	TG 44:0|TG 10:0_16:0_18:0
23	DG	DG 36:3|DG 18:1_18:2	47	TG	TG 44:1|TG 10:0_16:0_18:1
24	DG	DG 36:4|DG 18:2_18:2	48	TG	TG 44:2|TG 8:0_18:1_18:1
49	TG	TG 46:0|TG 14:0_16:0_16:0	89	TG	TG 56:6|TG 18:2_18:2_20:2
50	TG	TG 46:1|TG 12:0_16:0_18:1/TG 14:0_16:0_16:1	90	TG	TG 57:1|TG 16:0_23:0_18:1
51	TG	TG 46:2|TG 10:0_18:0_18:2	91	TG	TG 57:2|TG 16:0_23:0_18:2
52	TG	TG 48:0|TG 16:0_16:0_16:0	92	TG	TG 57:3|TG 21:0_18:1_18:2
53	TG	TG 48:1|TG 14:0_16:0_18:1/TG 16:0_16:0_16:1	93	TG	TG 58:1|TG 18:0_22:0_18:1/TG 16:0_24:0_18:1
54	TG	TG 48:2|TG 14:0_16:0_18:2	94	TG	TG 58:2|TG 22:0_18:1_18:1
55	TG	TG 48:3|TG 14:0_16:1_18:2/TG 12:0_18:1_18:2/TG 16:1_16:1_16:1	95	TG	TG 58:4|TG 22:0_18:2_18:2
56	TG	TG 50:0|TG 16:0_16:0_18:0	96	TG	TG 58:5|TG 22:1_18:2_18:2
57	TG	TG 50:1|TG 16:0_16:0_18:1	97	TG	TG 58:6|TG 22:1_18:2_18:3
58	TG	TG 50:2|TG 16:0_16:0_18:2	98	TG	TG 59:1|TG 16:0_25:0_18:1
59	TG	TG 50:3|TG 16:0_16:1_18:2/TG 14:0_18:1_18:2	99	TG	TG 59:2|TG 23:0_18:1_18:1/TG 16:0_25:0_18:2
60	TG	TG 50:4|TG 14:0_18:2_18:2/TG 16:1_16:1_18:2	100	TG	TG 59:3|TG 23:0_18:1_18:2
61	TG	TG 51:1|TG 16:0_17:0_18:1	101	TG	TG 59:4|TG 23:0_18:2_18:2
62	TG	TG 51:2|TG 16:0_17:0_18:2	102	TG	TG 60:1|TG 18:0_24:0_18:1/TG 16:0_26:0_18:1
63	TG	TG 51:3|TG 16:0_17:1_18:2	103	TG	TG 60:2|TG 24:0_18:1_18:1
64	TG	TG 51:4|TG 16:0_17:2_18:2/TG 15:0_18:2_18:2	104	TG	TG 60:3|TG 24:0_18:1_18:2
65	TG	TG 52:0|TG 16:0_18:0_18:0/TG 16:0_16:0_20:0	105	TG	TG 60:4|TG 24:0_18:2_18:2
66	TG	TG 52:1|TG 16:0_18:0_18:1	106	TG	TG 60:5|TG 24:1_18:2_18:2
67	TG	TG 52:2|TG 16:0_18:1_18:1	107	TG	TG 62:2|TG 26:0_18:1_18:1/TG 22:0_22:0_18:2
68	TG	TG 52:3|TG 16:0_18:1_18:2	108	TG	TG 62:3|TG 26:0_18:1_18:2
69	TG	TG 52:4|TG 16:0_18:2_18:2	109	TG	TG 62:4|TG 26:0_18:2_18:2
70	TG	TG 52:5|TG 16:1_18:2_18:2	110	TG	TG 62:5|TG 26:1_18:2_18:2
71	TG	TG 52:6|TG 16:2_18:2_18:2	111	OxTG	TG 43:2;2O|TG 16:0_15:2_12:0;2O
72	TG	TG 53:2|TG 17:0_18:1_18:1/TG 16:0_18:1_19:1	112	OxTG	TG 45:2;2O|TG 18:0_15:2_12:0;2O
73	TG	TG 53:3|TG 17:0_18:1_18:2	113	OxTG	TG 45:3;2O|TG 18:1_17:2_10:0;2O
74	TG	TG 53:4|TG 17:0_18:2_18:2	114	OxTG	TG 52:2;2O|TG 16:0_18:1_18:1;2O
75	TG	TG 53:5|TG 17:1_18:2_18:2	115	OxTG	TG 52:3;2O|TG 16:0_18:1_18:2;2O
76	TG	TG 54:0|TG 16:0_18:0_20:0/TG 16:0_16:0_22:0	116	OxTG	TG 52:4;2O|TG 16:0_18:2_18:2;2O
77	TG	TG 54:1|TG 16:0_20:0_18:1	117	OxTG	TG 54:2;2O|TG 16:0_19:2_19:0;2O
78	TG	TG 54:2|TG 18:0_18:1_18:1/TG 20:0_16:1_18:1	118	OxTG	TG 54:3;2O|TG 18:1_18:1_18:1;2O
79	TG	TG 54:3|TG 18:1_18:1_18:1	119	OxTG	TG 54:5;2O|TG 18:1_18:2_18:2;2O
80	TG	TG 54:4|TG 18:1_18:1_18:2	120	OxTG	TG 54:6;2O|TG 18:2_18:2_18:2;2O
			121	OxTG	TG 56:2;2O|TG 16:0_22:0_18:2;2O
81	TG	TG 54:5|TG 18:1_18:2_18:2	122	OxTG	TG 56:4;2O|TG 21:0_16:3_19:1;2O
82	TG	TG 54:6|TG 18:2_18:2_18:2	123	OxTG	TG 58:4;2O|TG 22:0_19:2_17:2;2O
83	TG	TG 54:7|TG 18:2_18:2_18:3	124	OxTG	TG 52:3;3O|TG 16:0_18:2_18:1;3O
84	TG	TG 56:1|TG 16:0_22:0_18:1/TG 18:0_20:0_18:1	125	OxTG	TG 54:4;3O|TG 18:1_18:2_18:1;3O
85	TG	TG 56:2|TG 16:0_22:0_18:2/TG 20:0_18:1_18:1	126	OxTG	TG 54:5;3O|TG 18:2_18:2_18:1;3O
86	TG	TG 56:3|TG 20:0_18:1_18:2	127	OxTG	TG 41:1;1O|TG 10:0_16:0_15:1;1O
87	TG	TG 56:4|TG 20:0_18:2_18:2	128	OxTG	TG 43:1;1O|TG 10:0_18:0_15:1;1O
88	TG	TG 56:5|TG 20:1_18:2_18:2	129	OxTG	TG 43:2;1O|TG 10:0_16:0_17:2;1O
130	OxTG	TG 43:3;1O|TG 10:0_18:2_15:1;1O	142	OxTG	TG 54:3;1O|TG 18:1_18:1_18:1;1O
131	OxTG	TG 45:2;1O|TG 10:0_18:1_17:1;1O	143	OxTG	TG 54:4;1O|TG 18:1_18:1_18:2;1O
132	OxTG	TG 45:3;1O|TG 10:0_18:1_17:2;1O	144	OxTG	TG 54:5;1O|TG 18:1_18:2_18:2;1O
133	OxTG	TG 45:4;1O|TG 10:0_18:1_17:3;1O	145	OxTG	TG 54:6;1O|TG 18:2_18:2_18:2;1O
134	OxTG	TG 45:5;1O|TG 10:0_18:2_17:3;1O	146	OxTG	TG 56:2;1O|TG 20:0_18:1_18:1;1O
135	OxTG	TG 50:1;1O|TG 16:0_16:0_18:1;1O	147	OxTG	TG 56:3;1O|TG 20:0_18:1_18:2;1O
136	OxTG	TG 50:2;1O|TG 16:0_16:0_18:2;1O	148	OxTG	TG 56:4;1O|TG 19:1_19:1_18:2;1O
137	OxTG	TG 50:3;1O|TG 16:0_18:2_16:1;1O	149	OxTG	TG 58:2;1O|TG 22:0_19:1_17:1;1O
138	OxTG	TG 52:2;1O|TG 16:0_18:1_18:1;1O	150	OxTG	TG 58:4;1O|TG 22:0_18:2_18:2;1O
139	OxTG	TG 52:3;1O|TG 16:0_18:1_18:2;1O	151	EtherTG	TG O-55:5|TG O-19:1_18:2_18:2/TG O-19:2_18:1_18:2
140	OxTG	TG 52:4;1O|TG 16:0_18:2_18:2;1O	152	EtherTG	TG O-59:3|TG O-19:1_18:2_22:0/TG O-19:2_18:1_22:0

## Data Availability

The data presented in this study are available on request from the corresponding author.
